# Impacts of Climate Change-Induced Temperature Rise on Phenology, Physiology, and Yield in Three Red Grape Cultivars: Malbec, Bonarda, and Syrah

**DOI:** 10.3390/plants13223219

**Published:** 2024-11-15

**Authors:** Deolindo L. E. Dominguez, Miguel A. Cirrincione, Leonor Deis, Liliana E. Martínez

**Affiliations:** 1 Cátedra de Fisiología Vegetal, Facultad de Ciencias Agrarias, Universidad Nacional de Cuyo (UNCuyo), Almirante Brown 500, Chacras de Coria, Mendoza 5507, Argentina; ddominguez@fca.uncu.edu.ar (D.L.E.D.); mcirrincione@fca.uncu.edu.ar (M.A.C.); ldeis@fca.uncu.edu.ar (L.D.); 2 Grupo de Fisiología Vegetal y Microbiología, Instituto de Biología Agrícola de Mendoza (IBAM), Consejo Nacional de Investigaciones Científicas y Técnicas, Facultad de Ciencias Agrarias, Universidad Nacional de Cuyo, Almirante Brown 500, Chacras de Coria, Mendoza 5507, Argentina

**Keywords:** viticulture, heat stress, phenological development, *Vitis*, heat waves

## Abstract

Climate change has significant implications for agriculture, especially in viticulture, where temperature plays a crucial role in grapevine (*Vitis vinifera*) growth. Mendoza’s climate is ideal for producing high-quality wines, but 21st-century climate change is expected to have negative impacts. This study aimed to evaluate the effects of increased temperature on the phenology, physiology, and yield of Malbec, Bonarda, and Syrah. A field trial was conducted over two seasons (2019–2020 and 2020–2021) in an experimental vineyard with an active canopy heating system (+2–4 °C). Phenological stages (budburst, flowering, fruit set, veraison, harvest), shoot growth (SG), number of shoots (NS), stomatal conductance (gs), chlorophyll content (CC), chlorophyll fluorescence (CF), and water potential (ψa) were measured. Additionally, temperature, relative humidity, light intensity, and canopy temperature were recorded. Heat treatment advanced all phenological stages by approximately two weeks, increased SG and NS, and reduced gs and ψa during the hottest months. CC and CF remained unaffected. The treatment also resulted in lower yields, reduced acidity, and increased °Brix in both seasons. Overall, rising temperatures due to climate change advance the phenological phases of Malbec, Syrah, and Bonarda, leading to lower yields, higher °Brix, and lower acidity, although physiological variables remained largely unchanged.

## 1. Introduction

Climate change is a phenomenon that has profound implications for agriculture, particularly in viticulture where temperature is crucial for grapevine growth and development. Consequently, rising temperatures are impacting grape yield, composition, and wine quality, leading to shifts in the traditional geography of wine production [1].

Various studies have examined the relationship between climate change and viticulture in regions like Oregon, California, and Europe, highlighting how high temperatures are reshaping the global viticultural landscape [2,3,4,5]. Specifically, Argentina’s future suitability for wine production will depend on temperature increases, water availability, and the frequency of extreme events [6]. Cooler climate regions, such as the Pampa region, might benefit, while the Atlantic sector will offer limited winemaking opportunities [3,7,8].

Furthermore, high temperatures significantly impact the phenology, physiological variables, and harvest parameters of red grapes [2]. For example, elevated temperatures can accelerate the phenological development of grapevine leading to early grape ripening [9,10,11,12,13,14]. This shift moves the ripening phase to hotter periods, which in turn alters grape composition. As a result, the quality of the grapes and wine may be compromised, as rapid sugar accumulation and acid degradation may occur faster than desired [15].

In addition, research has shown that both mean and maximum temperatures correlate with the timing of major phenological stages [16,17], suggesting that higher temperatures could decrease yields due to increased flower abortion [18].

Key stages, such as budbreak, flowering, and veraison, are all temperature-dependent, impacting both harvest timing and grape composition [19]. As temperatures rise, these stages may occur earlier, shortening critical periods in the grapevine lifecycle and altering the balance of sugars and acids, ultimately influencing wine quality.

Moreover, high temperatures affect key physiological variables of the vine, including photosynthesis rate and water use efficiency [20]. Excessive transpiration under extreme heat conditions can lead to water stress, affecting plant health and berry quality [12]. Higher temperatures can also induce stomatal closure, a water-conserving mechanism that limits gas exchange and potentially reduces photosynthesis and carbon assimilation [21,22]. Temperature fluctuations can further impact the chlorophyll content of plants, which affects photosynthetic capacity and overall vine health. Increased temperatures may lead to chlorophyll degradation or alter its synthesis rate, affecting light absorption efficiency and photosynthetic performance. Additionally, temperature stress can disrupt photosystem II (PSII) activity, as indicated by changes in chlorophyll fluorescence parameters like Fv/Fm (maximum quantum efficiency of PSII), signaling potential damage to the photosynthetic system [23,24,25].

Therefore, understanding the effects of temperature on parameters such as water potential, stomatal conductance, chlorophyll content, and chlorophyll fluorescence is essential for predicting and managing plant responses to changing environmental conditions.

The implications of rising temperatures on vineyards are complex, potentially decreasing yields and increasing pH levels in grapes, affecting sensory quality in wines. Consequently, grape growers need to adapt to these changes through practices like canopy management, irrigation adjustments, and selecting heat-tolerant varieties to mitigate the impacts of temperature increases [26,27,28,29,30,31]. Understanding and addressing these impacts is essential for the sustainability and resilience of viticulture. In this context, the objective of this study was to evaluate the effect of temperature increases on the phenology, physiological variables, and yield of Malbec, Syrah, and Bonarda cultivars.

## 2. Results and Discussion

### 2.1. Canopy Temperature

**Figure 1:**
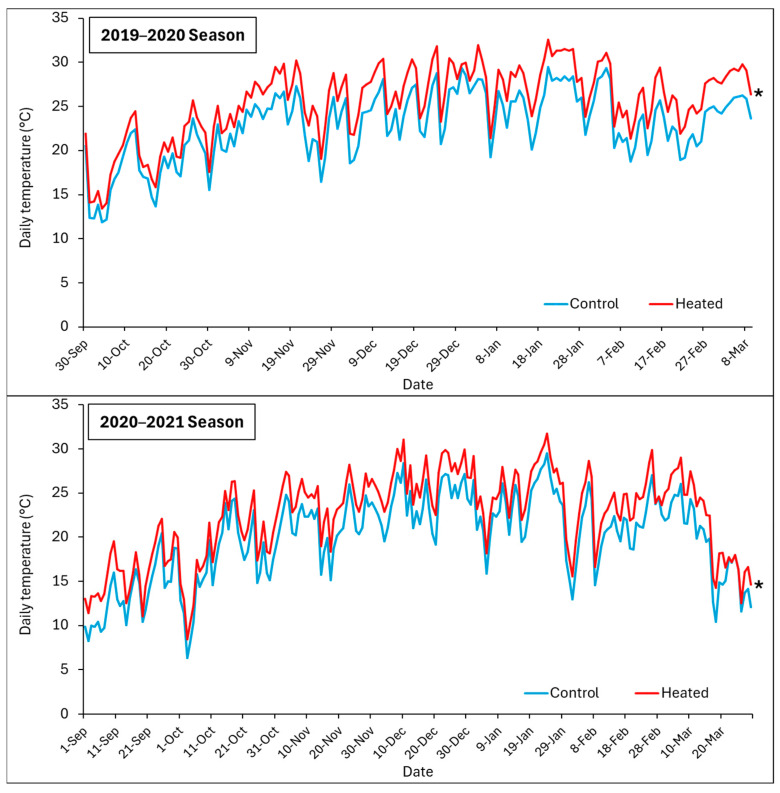
Average daily temperature within the canopy for the heated treatment (red lines) and control (blue lines) from October to March 2019–2020 (**top** panel), and October to March 2020–2021 (**bottom** panel).

Figure 1 shows the average daily temperature within the canopy for the heated treatment (+T) and control (−T) from September 2019 to March 2020 and from September 2020 to March 2021.

In terms of average daily temperature, +T consistently showed higher average daily temperatures compared to the control throughout the period from September 2019 to March 2020 and from September 2020 to March 2021. This indicated that +T maintained a consistently warmer environment compared to −T throughout the day, showing a sustained impact of +T on increasing temperatures within the canopy environment.

The meteorological data for the study years show relatively similar average temperatures, with the 2019–2020 season having an average of 21.5 °C and the 2020–2021 season slightly lower at 19.8 °C (Table 1). While the maximum temperatures were somewhat higher in the 2019–2020 season, with an overall maximum of 32.6 °C in January, the average temperature increase due to the treatment remained consistent across both years, with the canopy temperature being 2.5 °C higher than the ambient temperature. Additionally, precipitation was higher in the 2020–2021 season, with 228.2 mm compared to 131.1 mm in the 2019–2020 season. However, as shown in Figure 1, the temperature of the treatment remained higher than the control throughout the entire season, indicating that the system’s response was not significantly affected by the year-to-year variations in environmental conditions.

Although higher average daily temperatures could initially enhance physiological processes like photosynthesis, as grapevines generally benefit from warm temperatures up to a certain threshold, prolonged exposure to higher maximum temperatures could potentially lead to heat stress, affecting vine health and grape development negatively over time. Warmer minimum temperatures can impact nighttime recovery processes, potentially leading to increased respiration rates and altered metabolic activities.

Temperatures exceeding 35 °C can have various developmental, physiological, and biochemical effects on grapevines, influenced by interactions with other climate factors such as drought and wind, as well as their timing relative to the vine’s growth stages [23]. When temperatures rise above 40–45 °C, they can impair photosynthesis by damaging photosystem II and lead to irreversible leaf and berry scorching [32], significantly reducing fruit yield [31]. In this work, we confirmed the occurrence of temperatures over 40 °C for at least 8 days during both seasons.

Over the past few decades, rising temperatures have been linked to documented alterations in grapevine growth and physiological development [11,33,34,35].

### 2.2. Vine Physiology

**Figure 2:**
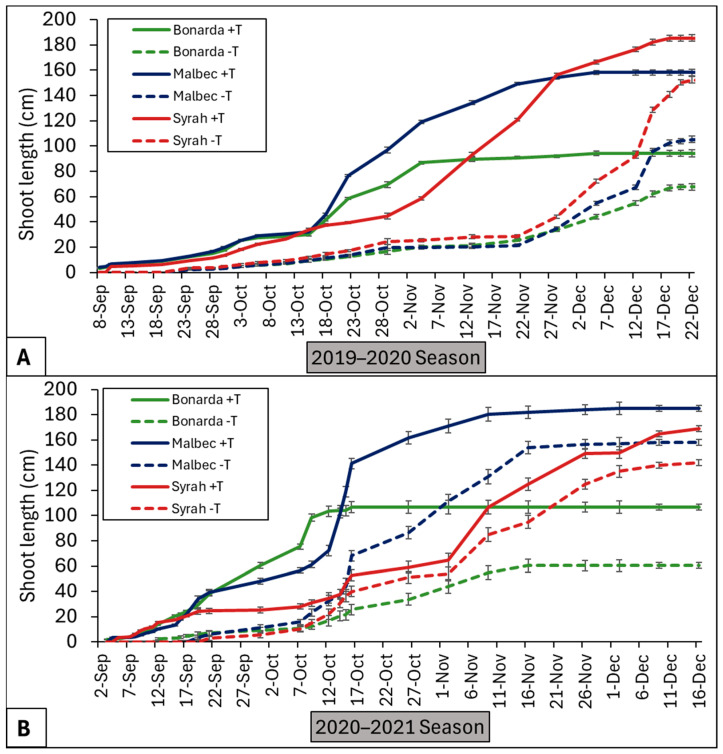
Cumulative apical shoot length (cm) under heated (solid lines) and control (dashed lines) treatments for Bonarda (green lines), Malbec (blue lines), and Syrah (red lines) from budbreak to constant length, in 2019–2020 (**A**) and 2020–2021 (**B**) seasons. Dots are average values of cumulative shoot length, and bars represent the standard error.

In this work, during growing season 2019–2020, Bonarda’s shoot length increased steadily for both treatments (Figure 2). However, the +T showed significantly higher growth than −T, especially towards the end of the vegetative growth stage. In terms of Malbec and Syrah, +T resulted in significantly greater shoot length than −T, with a particularly noticeable divergence starting from mid-October onwards. In the growing season 2020–2021, the three cultivars followed a similar pattern to the previous one (Figure 2). Warmer temperatures of thr +T treatment during early stages could have initially increased the photosynthesis rate because the temperatures were within the optimal range for grapevine C3 photosynthesis. These early temperature differences may have further amplified the effects of the +T treatment on photosynthesis and increased shoot growth by increasing the number of buds per shoot.

**Figure 3:**
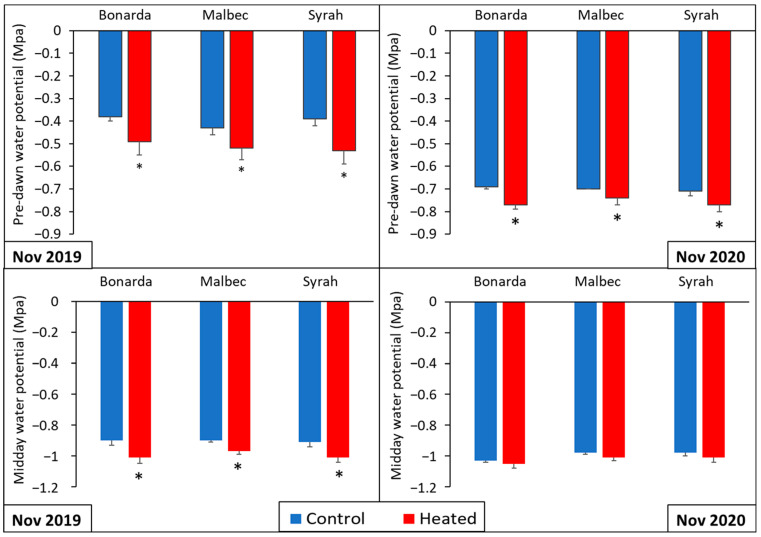
Predawn water potential (**upper panels**) and midday water potential (SWP) (**lower panels**) for the heated (red bars) and control (blue bars) treatments for Bonarda, Syrah, and Malbec. Measurements were carried out on 6 November 2019 (**left panels**) and 16 November 2020 (**right panels**). Bars represent means of three replicates ± standard error. Asterisks (*) in the heated bars indicate significant differences compared to the control at *p* < 0.05, according to the DGC test.

Figure 3 shows significantly lower predawn water potential values in +T compared to −T for Bonarda, Malbec, and Syrah. In both growing seasons, similar trends were observed with significantly lower predawn water potential values in +T for all cultivars. Similarly, midday water potential values were significantly lower in +T compared to the −T for all three cultivars, indicating higher water stress. In 2020, the same pattern persisted with significantly lower midday water potential values +T across all cultivars, reinforcing the observation of increased water stress. These results align with the findings of previous research, which demonstrated that vines of the same cultivars exhibited significantly lower leaf and stem water potential at 5:00 pm when they were exposed to natural heatwaves, a condition that could trigger xylem cavitation [31].

**Figure 4:**
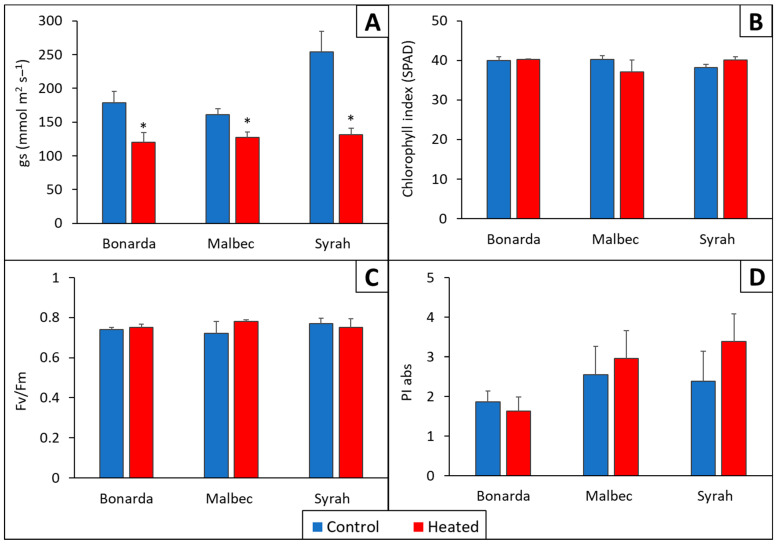
Stomatal conductance (gs) (**A**), chlorophyll index (SPAD) (**B**), maximum photochemical efficiency of Photosystem II (Fv/Fm) (**C**), and Photosynthetic Performance Index (PI) (**D**) for the heated treatments (red bars) and control (blue bars) for Bonarda, Syrah, and Malbec. The gs and SPAD measurements were made on 16 November 2020, while the Fv/Fm and PI measurements were conducted on 18 February 2021. Bars represent means of three replicates ± standard error. Asterisks (*) in the heated bars indicate significant differences compared to the control at *p* < 0.05, according to the DGC test.

Stomatal Conductance (gs) exhibited a significative decrease in vines of the three cultivars subjected to +T compared to −T during the 2019–2020 and 2020–2021 growing seasons (Figure 4). On average, Bonarda, Malbec, and Syrah +T showed a gs reduction of 32, 21, and 48%, respectively, compared to −T. This suggested that the vines under a higher temperature kept their stomata closed, which was likely to reduce water loss, while control vines could keep their stomata open, which probably led to a higher transpiratory rate. Similar findings were reported by [24,31], where midday gs was higher in vines treated with overhead spray water using aerial sprinkles that reduced leaf temperature compared to the control vines. In terms of chlorophyll index, Fv/Fm and PI, in this work, were not significantly different comparing +T and −T, suggesting that the vines did not affect their photosynthetic capacity, nor did they suffer from heat stress or photoinhibition.

**Figure 5:**
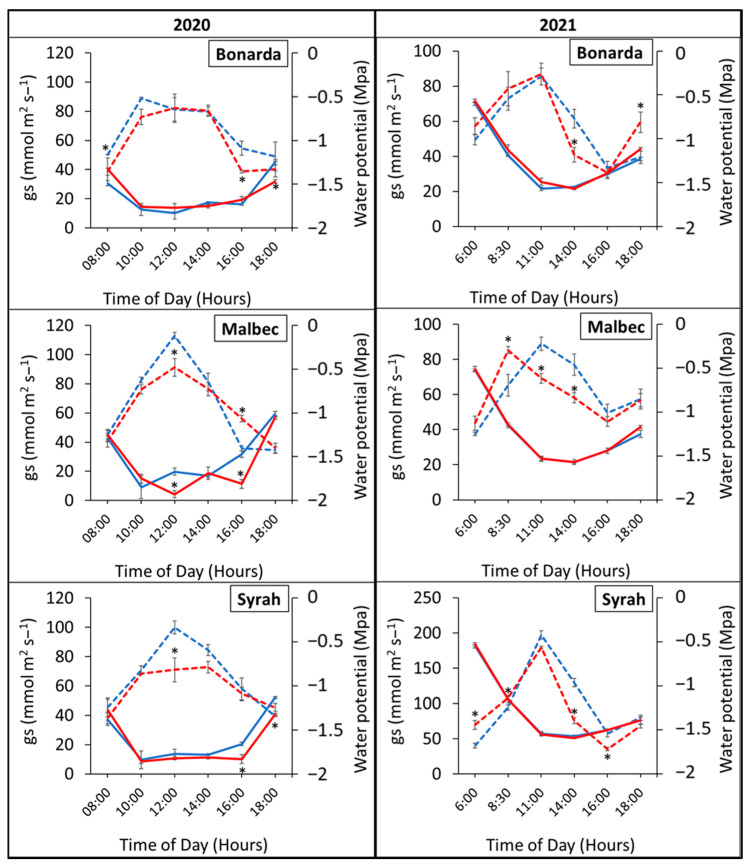
Daily evolution of stomatal conductance (gs) (dashed lines) and water potential (ψa) (solid lines) in the heated (red lines) and control (blue lines) treatments for Bonarda, Malbec, and Syrah. Measurements were taken on 21st February 2020—first growing season (**left panel**) and on 28th December 2020—second growing season (**right panel**). Each point represents the mean of three replicates ± standard error. Asterisks indicate statistically significant effects between treatments at *p* < 0.05.

Figure 5 illustrated the daily trends in stomatal conductance (gs) and water potential (ψa) across three grape varieties under both heated (+T) and control (−T) conditions. Stomatal conductance exhibited higher values under +T compared to −T, peaking at 10:00 h for Bonarda, and at 12:00 h for Malbec and Syrah. Probably, the high temperature treatment increased transpiration and water evaporation through the leaf surface, resulting in reduced water availability to the cells and subsequently lower water potential (ψa). The lower ψa under +T indicated that the plants were experiencing water stress due to increased water loss. The kinetics of gs along a day showed a negative correlation with water potential, indicating that as stomata began to open, stomatal conductance increased, leading to a decrease in water potential by transpiration water loss. This relationship persisted throughout the study period.

Stomatal conductance and water potential are key indicators of plant response to high temperatures [21]. Stomatal conductance decreases with increasing temperature and water stress, and water potential also decreases with thermal stress, reflecting a lower availability of water in the plants [22].

### 2.3. Changes in Phenology

**Figure 6:**
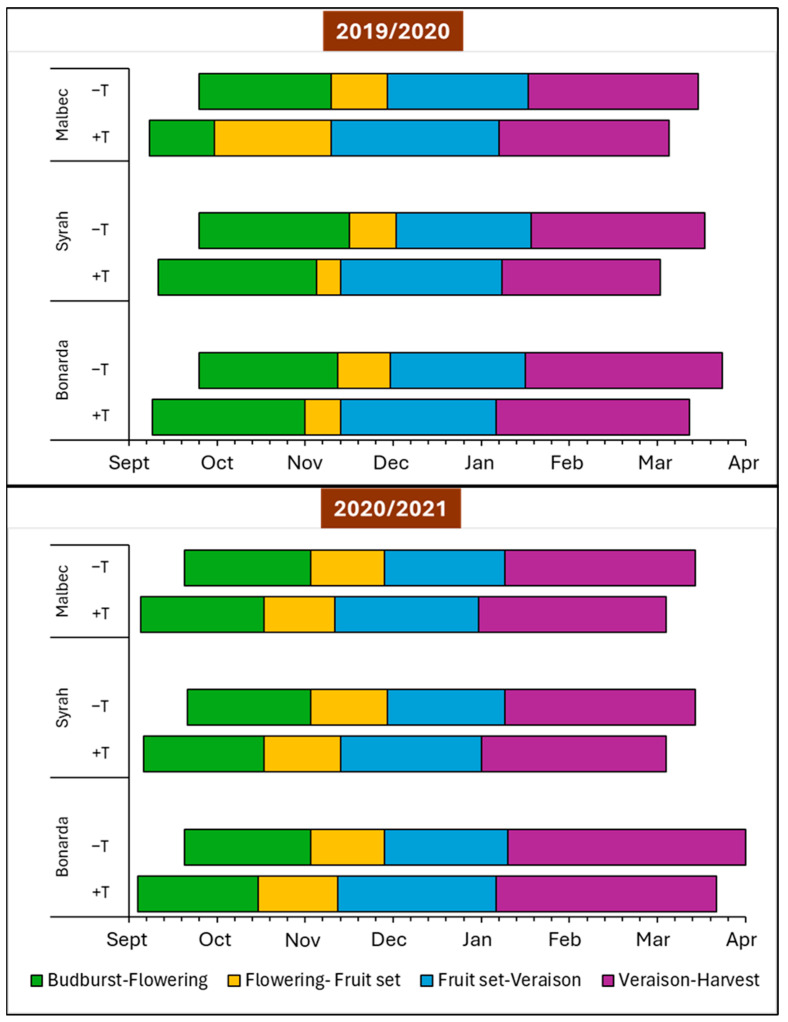
Phenological stages’ occurrence and duration in number of days for Malbec, Syrah, and Bonarda cultivars in both Heated and Control treatments in seasons 2019/2020 and 20120/2021. Each stage represents 50% of the clusters reaching the following periods: budburst, flowering, fruit set, veraison, and harvest.

Figure 6 highlighted the impact of the treatment condition on the phenological stages of Malbec, Syrah, and Bonarda grape cultivars in both seasons. Budbreak occurred earlier under +T condition by 2 weeks compared to −T in both seasons in all cultivars. Although the last spring frost date is occurring earlier around the world, accelerating the initial growth phase of grapevines through high temperature can still increase their risk of suffering from spring frost [1]. Furthermore, flowering and fruit set also occurred earlier under the treatment condition for all cultivars across both seasons. The advancement in these stages suggested that +T promoted faster flowering and quicker transition from flowering to fruit development. In addition, veraison, the stage when grapes begin to ripen, was consistently earlier, indicating accelerated ripening under +T for all cultivars in both seasons. The harvest dates exhibited a similar trend and were earlier with +T, causing grape ripening to occur in a warmer part of the summer, which can have undesired effects.

In summary, across all phenological stages and grape cultivars tested in this work, +T consistently leads to an earlier onset of all phenological stages compared to −T, by approximately 14 days. This pattern was observed consistently across both growing seasons (2019–2020 and 2020–2021) in the climate conditions of Mendoza, Argentina and were in accordance with those reported by [1], who described that elevated temperatures have been accelerating the progression of all phenological phases in most winegrowing regions around the globe over the past 40 years, leading to an advanced grape harvest by 2–3 weeks.

The duration of the different phenological stages varies significantly, not only according to each grape cultivar but also due to the thermal conditions for each specific year, and can be an important indicator to assess the impact of treatments on the physiology and development of grapes, which is crucial for viticultural production and crop management. As shown in Table 2, the growing season length (GSL) varied from 169 to 198 days in both seasons, depending on the year, cultivar, and treatment. There was no evidence for a change in the interval length of the growing cycle for Syrah during season 2019–2020. On the contrary, Malbec and Bonarda tended to extend it by 5–7 days under +T in both years. Bonarda exhibited the longest growing season in both growing seasons, attributed to its long-cycle nature as a cultivar. Regarding the phenological intervals (BVL and VHL) during 2019–2020, they were consistent across varieties and did not show marked differences between +T and −T. While in 2020–2021 +T augmented the interval of BVL, VHL did not show differences. Despite budbreak and flowering advancing by 15 days in Alsace, France from 1965 to 2003, the overall duration between these phenological stages remained unchanged [13]. Our results contradicted a previous study [36], which used climate models to predict that future high temperatures may shorten the overall duration of the grapevine growth cycle.

Thermal integrals (TIGSL, TIBVL, TIVHL) represent the accumulation of heat over different phenological periods, with higher values indicating greater heat accumulation, which can significantly influence grape development and ripening. Notably, our findings showed that +T resulted in a greater thermal accumulation of TIGSL, TIBVL, and TIVHL compared to −T during both years of study. This increased heat leads to an acceleration in grape development and maturation, extending the growing season slightly, and potentially impacting grape quality and wine characteristics.

### 2.4. Yield and Grape Composition

**Figure 7:**
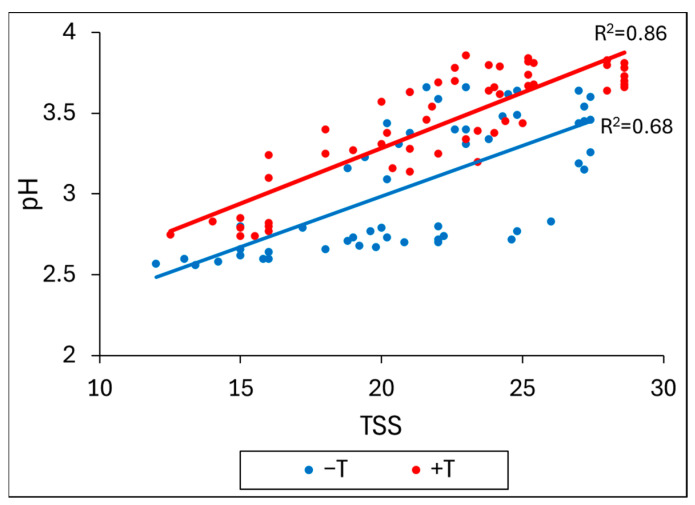
Correlation between total soluble solids (TSS) and pH from veraison dates to harvest in the heated treatment (red dots) and control (blue dots) for Bonarda, Malbec, and Syrah in the 2019–2020 season.

Figure 7 shows the correlation between total soluble solids (TSS) and pH from veraison to harvest dates during the 2019–2020 season for Bonarda, Malbec, and Syrah. Under +T treatment, the cultivars exhibited higher TSS and pH values across the dates measured, with high correlation (R^2^ = 0.86). In contrast, under −T treatment, the correlation was lower (R^2^ = 0.68). This indicates a more pronounced increase in TSS and pH with temperature increase. This difference could be attributed to the fact that during the maturation period, the thermal integral was, on average, higher for +T (956.05) than for −T (801.53). The results of the present work explain how higher temperatures accelerate grape maturation (Table 3) and shift ripening to a warmer part of the summer (Figure 6). This shift leads to increased pH mainly by malic acid degradation and sugar content in the grapes (Table 4), which ultimately results in higher alcohol levels and wine pH, along with decreased acidity. Similar findings were reported by other researchers [12,37,38], indicating that the changes caused by high temperature may potentially decrease wine quality.

Table 3 presented results for fruit set (%), number of clusters per plant, cluster weight (g), number of berries per cluster, berry weight (g), fruit yield (kg/plant) at harvest time, and Ravaz index for the seasons 2019–2020 and 2020–2021. Yield components during the growing season 2019–2020 experienced significant decreases due to +T. Specifically, the percentage of fruit set, cluster weight, number of berries per cluster, and fruit yield decreased by 43.2%, 19.1%, 13.6%, and 29.4%, respectively, compared to −T. However, there were no differences in the number of clusters or berry weight between +T vs. and −T. Additionally, the Ravaz index, which indicates vine balance and represents the relationship between fruit yield and pruning weight, decreased with +T by 22,78% (from 8.52 for −T vines to 6.76 for +T vines), indicating a more balanced vine for the former. Ref. [39] established an optimal range for his index, between 5 and 10, to indicate a balanced vine capable of achieving both fruit quality and consistent production.

When comparing the cultivars, Malbec showed the highest fruit set, number of clusters per plant, and fruit yield. Along with the Syrah cultivar, the cluster weight and number of berries per clusters were significantly higher compared to Bonarda. Conversely, Bonarda showed the highest berry weight in both seasons. The lowest Ravaz index was shown by Malbec, followed by Syrah and Bonarda.

Fruit set was the variable that exhibited interaction between treatment and cultivar, being statistically lower for +T compared to −T for the three cultivars in both years. In terms of the Ravaz index, Temp × Malbec and Temp × Syrah showed the lowest value relative to their respective −T vines, indicating more vegetative plants compared to Bonarda, which presented highly productive vines.

Growing season 2020–2021 confirmed the results of the previous 2019–2020 season, showing the same significant reduction for all the variables, including the number of clusters per plant, in +T compared to −T vines. Malbec also showed the highest values of fruit set, number of clusters per plant, berry weight, and yield compared to Syrah and Bonarda. Again, Bonarda showed the highest value for berry fresh weight.

These findings demonstrate that the elevated temperatures resulting from the active heating treatment, particularly during November when fruit set occurs in the Southern Hemisphere, caused a reduction in the fruit set percentage. Consequently, although berries presented the same weight comparing +T and −T, there were a significative lower number of them at harvest, leading to lighter clusters. This ultimately resulted in diminished yields for the plants subjected to the temperature treatment by 25.85% in comparison to the control ones in both growing seasons.

Numerous studies have evaluated the impact of heating on yield and have hypothesized that losses could be even more severe when high temperatures, due to heatwaves, occur during or immediately after flowering, causing flower abortion and a reduction in bunch biomass [25,40]. Yield losses ranging from 24 to 45% have been well documented during increasing temperatures due to heatwaves [31,32,41]. Nevertheless, according to [16], as the springtime maximum temperatures increase, the effect on this phenological stage could be not uniform and may depend on the cultivar and region, making it necessary for viticulturists to tailor to their individual situation.

Table 4 shows results for total soluble solids (TSS) in °Brix and pH levels across +T and −T, cultivars (Malbec, Syrah, Bonarda), and their interactions for the growing seasons 2019–2020 and 2020–2021.

During the first harvest in 2020, average TSS was significantly higher in +T (25.78 °Brix) than in −T (24.28 °Brix). In terms of cultivars, Syrah showed the highest TSS (27.80 °Brix), followed by Malbec (24.68 °Brix), while Bonarda had the lowest (22.60 °Brix). In 2021 average TSS was 23.87 °Brix for +T, which was again significantly higher than −T (23.29 °Brix). Syrah again showed the highest TSS (25.83 °Brix), followed by Malbec (24.30 °Brix), and Bonarda had the lowest (20.47 °Brix). Across both seasons, +T consistently resulted in higher TSS levels compared to −T, indicating that the treatment induced a significantly higher sugar accumulation in the grapes. However, pH levels did not show any significative differences between treatment, cultivars, and their interaction.

Different grape cultivars exhibited significant variability in both TSS and pH levels across the years. Syrah generally showed higher TSS and lower pH compared to Malbec and Bonarda, reflecting inherent varietal differences in sugar content and acidity.

While trends in TSS and pH were consistent between years under the same treatment, slight variations in average values and significance levels highlight potential yearly climatic influences on grape composition.

In previous works, it was found that higher temperatures during the growing season promoted a decrease in the grape berry total acidity content [42,43], upward trends in sugar content [29], and a decoupling between technological and phenolic maturity [44]. An increase in soluble solid content would likely lead to more alcoholic wines, as noted by [11,45], who observed that higher temperatures not only advance the harvest date but also increase alcohol levels in many regions, such as Bordeaux and Alsace in France. Moreover, extreme events during the veraison–maturity period, such as heatwaves, can significantly influence sugar accumulation [46].

### 2.5. Overall Perspectives

Climate change has a two-fold impact on the ripening period due to (i) generally increasing air temperatures and (ii) the shift of the ripening period toward earlier, typically warmer parts of the season. This effect is particularly evident during the ripening period in recent years [47]. Understanding these temperature variations is crucial for assessing the impact of the heated treatment on grapevine physiology and overall vineyard performance.

Insights into the response of grapevine phenology both at vineyard level and for different cultivars will further our ability to adapt and help to identify techniques to delay or ameliorate these climate-induced changes. The advancement of phenological phases is negatively affecting the grape composition at harvest time. Overall, the acceleration, likely due to higher temperatures, suggests significant impacts of climate change on grapevine growth and development, changing wine quality and style and thus emphasizing the need for adaptive viticulture strategies. It is important to highlight that all analyzed cultivars, Malbec, Bonarda, and Syrah, showed significant effects on all measured variables, demonstrating their collective vulnerability to increasing temperatures due to climate change. As additional information, Bonarda was the most affected under +T, as it exhibited the lowest fruit set percentage in both years, resulting in lower yields. Moreover, in the study by Wilson et al. (2024), it was observed that Bonarda increased the percentage of glycosylated anthocyanins while decreasing that of acetylated and coumarylated forms, further highlighting its sensitivity during heat waves.

Climate change implies that vineyards are increasingly subjected to constraining climate conditions, such as elevated temperature and heatwaves, which would be more frequent and stronger in amplitude along with altered precipitation patterns [1,31,48,49]. The irrigation dose applied in our study aligns with typical vineyard practices. However, further research will be essential to assess the combined effects of rising temperatures and varying irrigation frequencies and amounts. Adjustments in irrigation strategies may ultimately be necessary to sustain vine health and productivity under changing climate conditions.

This study illustrates that the simulated temperature increase, reflecting the IPCC predictions for the end of the century in Mendoza province, will result in adverse impacts. These include the accelerated advancement of all phenological phases, alterations in various physiological parameters, reduced yield accompanied by higher sugar concentration, and lower berry acidity levels. Furthermore, there will be a decrease in plant reserve accumulation.

## 3. Materials and Methods

### 3.1. Experimental Site

The experiment was conducted in an experimental vineyard at the Faculty of Agricultural Science of the National University of Cuyo located in Luján de Cuyo in Mendoza province in western Argentina (33°00′30.2″ S and 68°52′20.9″ W). Eight-year-old own-rooted *Vitis vinifera* cultivars—Malbec (ML) (clone N° 2), Syrah (SY) (clone N° 84), and Bonarda (BO) (clone N° 9)—were used for the experiment. Vines were spaced at 1 m intervals within rows and 2.2 m between rows, all oriented north–south, and were managed with spur pruning, employing a fruit load adjustment tailored to the individual vigor of each plant, utilizing a bilateral cordon system with shoots vertically positioned.

Prior to the budbreak, flowering, fruit set, and post-veraison stages, all vines were drip irrigated for 48 h. The drip distance was 1 m, and the water application rate was 2 L h^−1^. Air temperature was measured by two temperature sensors (iButton 1 Wire^®^ Thermochron^®^ Maxim Integrated USA, San Jose, CA, USA) sheltered inside plastic boxes avoiding direct sun exposure and installed at either end of each row. The experiment was performed during the growing seasons 2019–2020 and 2020–2021 from budbreak until harvest (September to March). Table 1 provides details on environmental data at the experimental site for the 2019–2020 and 2020–2021 seasons.

### 3.2. Experimental Design

**Figure 8:**
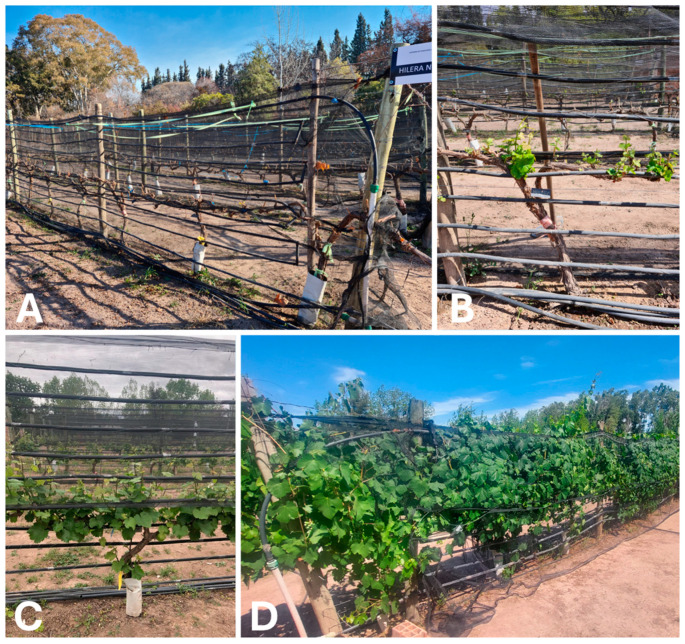
Vineyard heating system at different phenological stages of the vines. (**A**) Before budbreak. (**B**) Shoots about 10 cm long. (**C**) Shoot development stage. (**D**) Fully developed vine.

The experimental design consisted of a randomized plot of six rows (three rows for treated temperature and three rows for control), with six plants per row. Each row contained two plants of each cultivar (Malbec, Syrah, and Bonarda). Three replicates of each treatment were used. Treatment consisted of an active heating system (+T) that successfully created a controlled temperature rise within the range predicted by the SSP2–4.5 scenario, where the mean temperature in this region is projected to increase by 1.5 °C to 2.5 °C by the end of this century [49]. All aspects of the active heating system functionality are detailed by [50]. Briefly, the system uses electric hot water tanks and polypropylene pipes integrated into the vineyard structure. The system operates by circulating water heated to 60 ± 1 °C through pipes connected to flexible polyethylene tubes placed along the vine rows (Figure 8). These tubes are arranged in a serpentine shape on the support structures of the plants, allowing heat to be evenly distributed around the canopy. The water, after releasing its heat, returns to the tanks in a closed loop, where it is reheated to continue the process. This active system can precisely control the increase in air temperature around the plants, achieving average temperature increases of 2.5 ± 0.12 °C, regardless of weather conditions, unlike passive systems that rely on solar radiation. In contrast, control treatment was not heated (−T). The treatment +T started 1 months before sprouting and extended until the harvest.

Phenological stages were determined at the onset of budbreak, flowering, fruit set, veraison, and harvest in both seasons for the three cultivars under heat (+T) and control (−T).

### 3.3. Vegetative and Physiological Measurements

The shoot length (cm) and the number of buds were assessed from budbreak in vines under treatment and control in the three cultivars from September to December in both years.

Monthly, leaf water potential (LWP) was measured at 5 am (pre-dawn), 8 am, 11 am, 2 pm and 5 pm in both growing seasons. Completely healthy, dry and mature leaves were randomly selected from the middle of the canopy at the first training wire for measurement. Each leaf was placed in a plastic bag and sealed prior to removal by cutting the petiole with a razor blade. Leaves were immediately placed in a pressure chamber operated in the field (Model 4, Biocontrol) to assess water potential. Stem Water Potential (SWP) was measured at midday (Mendoza, South Hemisphere) from 14:00 to 15:00 after the intact leaves had been covered with aluminum foil for 30 min prior to leaf removal and measurement.

The relative chlorophyll content (CC) of leaves was measured with the SPAD-502 Plus, (Konica Minolta, Osaka, Japan) in duplicate for each replicate at 11 am and 5 pm. Stomatal conductance (gs) (SC1 Leaf Porometer, Decagon Devices, Pullman, WA, USA) was measured on leaves four times per day at 8 am, 11 am, 2 pm, and 5 pm in both years during the growing season. Chlorophyll fluorescence measurements (Fv/Fm ratio and Performance Index on absorption basis (PI abs)) were carried out after 20 min of dark exposure at 11 am and 5 pm.

The evolution of stomatal conductance and water potential was also evaluated at different times during both growing seasons.

### 3.4. Inflorescence Set, Fruit Yield, Ravaz Index, Soluble Solids Contents and pH

To measure the percentage of inflorescence set, each treated shoot per plant was marked, and a tulle bag was placed over the basal inflorescence of each shoot in both seasons, from the beginning of flowering [51] until one month after set, to collect fallen calyptras and un-set ovaries. The analysis was conducted manually as described by [52]. The contents of the bags were poured onto a white surface, and the calyptras were separated from the aborted ovaries. Both were counted, and the percentage of aborted ovaries was calculated by the ratio of the number of un-set ovaries to the number of flowers in the inflorescence × 100. The percentage of set (proportion of flowers that set a berry) was calculated after harvest as the ratio of the number of flowers in the inflorescence to the final number of berries harvested from the marked cluster × 100.

The clusters were harvested when the total soluble solids (TSS) of −T reached 24 °Brix ± 2. They were measured by refractometry (°Brix) (Atago^®^, Master–T Japan, Kyoto, Japan), and pH using a pHmeter (Altronix^®^, New York, NY, USA). Cluster fresh weight (FW) and number of berries per cluster were determined. Total berries fresh weight and number of berries were recorded.

The Ravaz index was used to evaluate the balance between grape yield and vine vigor. It was calculated as the ratio of the weight of the fruit (fruit grape yield) to the weight of the vine’s pruning wood (vegetative growth), according to the next formula:

Ravaz index = Yield (kg/vine)/Pruning weight (kg/vine)

The calculation of growing degree days was carried out for GDD in base 10 °C as follows:

$$GDD_{x}=\Sigma_{i=1\;}^{n}(T{}^{\circ}mean,i-x)$$

where *T°mean* represents the average daily temperature and *x* the temperature threshold (i.e., 10 °C). For this work, we used GDD_10_ as an indicator of early thermal accumulation in grapevines.

### 3.5. Statistical Analysis

Results were subjected to a two-way ANOVA to examine the effects of the treatments, cultivars, and treatment and cultivar interaction. The Shapiro–Wilk and Levene Tests were performed to determine normality and homoscedasticity. Data are presented as means of three replicates (n = 3). Mean comparisons were performed by Tukey test, considering *p* ≤ 0.05 (*) significant, *p* ≤ 0.01 (**) highly significant, *p* ≤ 0.001 (***) very highly significant, and ‘NS’ not significant.

Rates of shoot elongation, predawn water potential, and stem water potential were analyzed as repeated measurements in time, using analysis of variance (ANOVA) by mixed linear models with a factorial structure, treating heated treatment, cultivar, and their interactions as fixed effects, while replicates were treated as random effects. Means comparisons were performed using the DGC test [53]. Data are presented as means ± standard error (SE) of three single-plant replicates. Pearson correlation analysis was conducted to test for relationships between pH and TSS. Statistical analysis was performed with the software InfoStat version 2020 [54], R^®^ version 3.5.3 (R Core Development Team, Vienna, Austria) and XLSTAT-Pro statistical package [55].

## 4. Conclusions

In conclusion, this study demonstrates that a simulated temperature increase (+T) has a significant impact on the phenological and physiological development of the Malbec, Bonarda, and Syrah grapevine cultivars, as well as on grape yield and quality. The results show that vines under +T experienced an acceleration in all phenological stages, from budbreak to ripening, leading to earlier harvests and maturation under warmer conditions. This acceleration negatively affected grape composition, resulting in higher sugar concentration and lower acidity, which could impact wine quality by increasing alcohol levels and reducing freshness. Additionally, reduced yield, due to lower fruit set and lighter clusters, highlights the adverse effects of elevated temperatures on grapevine productivity.

These findings align with previous studies linking climate change to the advancement of phenological phases and the alteration of plant physiology, affecting both growth and final wine quality. In this context, the need for adaptive viticultural strategies becomes evident to mitigate the effects of global warming. Strategies such as proper irrigation management and techniques to delay ripening or protect plants from thermal stress are essential to maintain vineyard sustainability and wine quality in the future.

## Figures and Tables

**Table 1:** Temperature and precipitation data for September to October in the 2019–2020 and 2020–2021 seasons in Chacras de Coria, Luján de Cuyo, Mendoza, Argentina.

Season	Environmental Variable	Sep	Oct	Nov	Dec	Jan	Feb	Mar	Whole Period
2019–2020	mean maximum temperature (°C)	21.3	26.2	29.7	31.6	32.6	29.5	32.3	29.0
	mean average temperature (°C)	14.4	19.8	21.5	23.7	25.0	21.8	24.4	21.5
	mean minimum temperature (°C)	5.6	13.3	14.1	15.0	16.9	14.8	17.0	13.8
	accumulated precipitation (mm)	0.0	3.8	15.7	9.7	37.6	64.3	0.0	131.1
2020–2021	mean maximum temperature (°C)	18.6	24.3	28.2	31.2	29.8	28.0	26.2	26.6
	mean average temperature (°C)	11.3	16.1	22.4	25.0	23.0	22.2	18.7	19.8
	mean minimum temperature (°C)	4.7	7.6	16.0	16.3	15.8	14.0	12.0	12.3
	accumulated precipitation (mm)	0.0	0.0	24.8	3.3	54.0	86.7	59.4	228.2

**Table 2:** Growing season length (GSL) (days), flowering–veraison interval length (BVL) (days), veraison–harvest interval length (VHL) (days), thermal integral of the growing season length (TIGSL), thermal integral of the flowering–veraison interval length (TIBVL), thermal integral of veraison–harvest interval length (TIVHL) during seasons 2019–2020 and 2020–2021 of Malbec, Syrah, and Bonarda cultivars under Treatment (+T) and Control (−T).

Season	Cultivar	Treatment	GSL (Days)	BVL (Days)	VHL (Days)	TIGSL	TIBVL	TIVHL
2019–2020	Malbec	+T	176	67	57	2440.78	1090.84	966.59
2019–2020	Malbec	−T	169	67	57	2063.63	950.51	764.66
2019–2020	Syrah	+T	171	64	54	2395.66	1060.73	821.38
2019–2020	Syrah	−T	171	62	58	2085.75	887.67	771.2
2019–2020	Bonarda	+T	182	65	65	2529.70	1064.00	1080.20
2019–2020	Bonarda	−T	177	64	66	2152.11	908.79	868.72
2020–2021	Malbec	+T	180	72	65	2464.37	1068.22	949.91
2020–2021	Malbec	−T	175	66	66	2000.81	852.53	771.02
2020–2021	Syrah	+T	179	72	65	2454.87	1068.22	949.91
2020–2021	Syrah	−T	174	66	66	1993.81	852.53	771.02
2020–2021	Bonarda	+T	198	75	81	2667.84	1108.49	1127.05
2020–2021	Bonarda	−T	192	67	82	2169.79	865.77	926.76

Values are expressed as average (n = 3). Tukey test *p* ≤ 0.05, *p* ≤ 0.01, *p* ≤ 0.001.

**Table 3:** Fruit set, number of clusters per plant, cluster weight (g), number of berries per cluster, berry weight (g), fruit yield (kg/plant) at harvest, and Ravaz index in the season 2019–2020 and 2020–2021.

Year 2020														
Treatment	Fruit Set (%)		N° of Clusters		Cluster Weight (g)		N° of Berries		Berry Weight (g)		Yield (kg/plant)		Ravaz Index	
+T	29.03	b	65.56		66.71	b	53.75	b	1.26		3.12	b	6.76	b
−T	50.42	a	65.22		82.30	a	62.19	a	1.32		4.42	a	8.52	a
*p*-value	<0.0001	***	0.936	NS	0.0001	***	0.014	*	0.276	NS	<0.0001	***	0.0001	***
Cultivar														
Malbec	48.45	a	80.33	a	80.49	a	60.83	a	1.25	b	5.05	a	4.25	c
Syrah	38.46	b	64.00	b	77.84	a	60.79	a	1.09	c	3.29	b	5.70	b
Bonarda	32.26	c	51.83	c	65.51	b	50.79	b	1.54	a	2.97	b	12.97	a
*p*-value	<0.0001	***	0.0001	***	0.003	**	0.011	*	<0.0001	***	<0.0001	***	<0.0001	***
Treatment × Cultivar														
+T × Malbec	37.34	cd	81.67		77.10		59.18		1.29		4.60		2.85	d
−T × Malbec	59.57	a	79.00		83.83		62.50		1.20		5.50		5.64	bc
+T × Syrah	39.90	d	65.33		56.01		54.42		1.07		2.53		4.09	cd
−T × Syrah	43.01	bc	62.67		75.01		67.17		1.10		4.05		7.30	bc
+T × Bonarda	15.85	e	49.67		67.02		47.67		1.43		2.24		13.37	a
−T × Bonarda	48.67	b	54.00		86.67		53.92		1.65		3.70		12.61	a
*p*-value	<0.0001	***	0.727	NS	0.13	NS	0.346	NS	0.068	NS	0.319	NS	<0.0001	***
**Year 2021**														
**Treatment**	**Fruit Set (%)**		**N° of Clusters**		**Cluster Weight (g)**		**N° of Berries**		**Berry Weight (g)**		**Yield (kg/plant)**		**Ravaz Index**	
+T	34.21	b	79.67	b	49.84	b	60.22	b	0.86	a	4.01	b	6.62	b
−T	61.53	a	86.89	a	59.57	a	70.39	a	0.89	a	5.16	a	7.75	a
*p*-value	<0.0001	***	0.004	**	<0.0001	***	<0.0001	***	0.265	NS	<0.0001	***	0.003	***
Cultivar														
Malbec	60.43	a	109.67	a	53.27	b	60.00	b	0.89	b	5.96	a	4.39	b
Syrah	44.59	b	75.17	b	48.01	c	81.00	a	0.59	c	3.72	b	5.13	b
Bonarda	38.58	c	65.00	c	62.83	a	54.92	b	1.14	a	4.07	b	12.03	a
*p*-value	<0.0001	***	<0.0001	***	<0.0001	***	<0.0001	***	<0.0001	***	<0.0001	***	<0.0001	***
Treatment × Cultivar														
+T × Malbec	46.34	c	105.67		49.04		55.75		0.88		5.55	ab	3.18	c
−T × Malbec	74.52	a	113.67		57.5		64.25		0.9		6.38	a	5.61	b
+T × Syrah	36.54	d	70.00		43.17		73.25		0.59		3.34	cd	3.81	c
−T × Syrah	52.65	bc	80.33		52.85		88.75		0.6		4.11	cd	6.46	b
+T × Bonarda	19.74	e	63.33		57.3		51.67		1.11		5.00	b	12.88	a
−T × Bonarda	57.43	b	66.67		68.36		58.17		1.18		3.13	d	11.18	a
*p*-value	0.001	**	0.395	NS	0.619	NS	0.306	NS	0.676	NS	0.018	**	0.0001	***

Values are expressed as average (n = 3). Different letters within the same column indicate significant differences among Treatment (+T, −T), Cultivar (Malbec, Syrah and Bonarda) and Treatment × Cultivar interaction. Tukey test *p* ≤ 0.05 (*), *p* ≤ 0.01 (**), *p* ≤ 0.001 (***), and NS indicates not significant.

**Table 4:** Content of total soluble solids (°Brix) and pH of in the season 2019–2020 and 2020–2021.

	Year 2020				Year 2021			
Treatment	TSS (°Brix)		pH		TSS (°Brix)		pH	
+T	25.78	b	3.81	b	23.87	b	3.61	b
−T	24.28	a	3.62	a	23.29	a	3.49	a
*p*-value	<0.0001	***	0.000	***	<0.0001	***	0.030	**
Cultivar								
Malbec	24.68	b	3.73		24.30	a	3.63	
Syrah	27.80	a	3.70		25.83	b	3.47	
Bonarda	22.60	c	3.72		20.47	c	3.54	
*p*-value	<0.0001	***	0.746	NS	<0.0001	***	0.059	NS
Treatment × Cultivar								
+T × Malbec	25.27		3.82		24.67		3.66	
−T × Malbec	24.10		3.64		23.93		3.59	
+T × Syrah	28.40		3.81		26.07		3.59	
−T × Syrah	27.20		3.59		25.6		3.35	
+T × Bonarda	23.67		3.82		20.07		3.53	
−T × Bonarda	21.53		3.63		20.87		3.56	
*p*-value	0.200	NS	0.877	NS	0.612	NS	0.200	NS

Values are expressed as average (n = 3). Different letters within the same column indicate significant differences among Treatment (+T, −T), Cultivar (Malbec, Syrah and Bonarda) and Treatment × Cultivar interaction. Tukey test *p* ≤ 0.01 (**), *p* ≤ 0.001 (***), and NS indicates not significant.

## Data Availability

Data is available on request.
